# Supplementary material for: Typhoid intestinal perforation in Francophone Africa, a scoping review
Source: PLOS Glob Public Health. 2024 Mar 29;4(3):e0003056. doi: 10.1371/journal.pgph.0003056 (PMC10980251; doi:10.1371/journal.pgph.0003056)
Supplement: S1 Table — (DOCX) [file pgph.0003056.s002.docx]

**S1 Table: Collated data on TIP from all included studies**

| Country of Study | Author | Study period | Age years (average) | Number of TIP (% peritonitis) | Postop complications (%) | Postop Mortality (%) | Ileostomy creation (%) |
| --- | --- | --- | --- | --- | --- | --- | --- |
| Benin | Lo Sorto et al (29) | 2002 - 2003 | 4 - 65 (12) | 44* | 17 (49%) | 13 (30%) | 0 |
|  | Allode et al(31) | 2010 - 2014 | 2 - 68 (23) | 64 (35%) |  |  |  |
|  | Caronna et al (32) | 2011 - 2012 | 2 - 75 (18) | 104* | 16 (15%) | 26 (31%) | 2 (2%) |
|  | Tobome et al(33) | 2018 - 2019 | 2 - 75 (23) | 40 (63.5%) |  |  |  |
|  | Sambo et al(30) | 2015 - 2016 | 4 - 71 (20) | 28 (53%) |  | 2 (7%) | 4 (14%) |
| Burkina Faso | Zida et al (36) | 2006 - 2008 | 14 - 68 (26) | 78* |  |  | 62 (79%) |
|  | Ouedraogo et al (34) | 2010 - 2014 | 2 - 56 (14) | 216 (43%) | 156 (72%) | 37 (17%) | 32 (15%) |
|  | Kambire, et al (37) | 2012 | 1 - 75 (30) | 40 (27%) |  | 6 (15%) |  |
|  | Ouedraogo et al (35) | 2014 - 2018 | 2 - 56 (16) | 212* |  | 21 (10%) | 49 (23%) |
|  | Kambire et al (38) | 2016 | 6 - 77 (19) | 29 (20%) | 10 (35%) | 4 (14%) | 5 (17%) |
| Cameroon | Chichom-Mefire et al(39) | 2007 - 2013 | 3 - 70 | 43 (14%) | 25 (58%) | 16 (37%) |  |
|  | Johnson Alebeleye et al (40) | 2017 - 2018 | 12 - 55 (24) | 38* |  | 6 (16%) | 4 (11%) |
|  | Bang et al(41) | 2019 - 2020 | (38) | 17 (14%) |  |  |  |
| Central African Republic | Bobossi Serengbe et al (42) | 1997 - 1998 | 10 month - 15 | 31* | 5 (16%) | 9 (29%) | 0 |
| Chad | Choua et al (43) | 2007 - 2012 | 15 - 70 (26) | 72 (15%) |  | 8 (11%) | 33 (46%) |
| Cote d’Ivoire | Kouame, et al (45) | 1990 - 2000 | 3 - 16 (9) | 48* | 22 (46%) | 3 (6%) | 3 (6%) |
|  | Kouame et al(44) | 1995 - 1993 | 5 - 64 (34) | 64* | 59 (92%) | 22 (34%) | 33 (52%) |
|  | Kouassi et al(46) | 1997 - 2002 | 4 - 69 (21) | 82* |  | 15 (18%) | 35 (43%) |
| DRC | Guy et al(47) | 2004 - 2015 | (20) | 39 (35%) | 31 (80%) | 5 (13%) | 5 (13%) |
| Guinea | Mallick & Klein (48) | 1993 - 1998 | 4 - 41 (20) | 7* |  | 1 (14%) | 0 |
| Mali | Togo et al(49) | 1999 - 2008 | 2 - 65 (15.2) | 385 (32%) | 83 (22%) | 34 (8%) | 59 (15%) |
|  | Sanogo et al (50) | 2000 - 2007 | (23) | 120* |  | 19 (16%) | 35 (29%) |
|  | Coulibaly et al (51) | 2005 - 2010 | 3 - 14 (10) | 105* | 39 (37%) | 16 (15%) | 31 (30%) |
| Niger | Harouna et al(52) | 1995 - 1996 | 4 - 60 (20) | 56 (35%) | 26 (46%) | 16 (29%) | 2 (4%) |
|  | Sani et al(55) | 2003 - 2006 | 6 - 65 (16) | 177 (42%) |  | 32 (18%) |  |
|  | Adamou et al (53) | 2013 - 2014 | 0 - 80 (21) | 174 (54%) |  |  |  |
|  | Magagi et al(56) | 2013 - 2014 | 0 - 95 (23) | 175 (55%) | 79 (45%) | 27 (15%) | 119 (68%) |
|  | Adamou et al (54) | 2013 - 2015 | 0 - 15 (10) | 153 (68%) |  | 22 (14%) | 96 (63%) |
|  | Adamou et al(21) | 2013 - 2019 | 1 - 70 (11) | 2931 (69%) | 907 (31%) | 328 (11%) | 1797 (61%) |
| Rwanda | Mutabazi et al (57) | 2015 - 2016 | 1m - 15 | 11 (17%) | 10 (91%) | 3 ( 27%) |  |
| Togo | Saxe & Cropsey (58) | 2003 | 3 - 75 (20) | 112* |  | 18 (16%) |  |
|  | Kassegne et al (59) | 2009 - 2011 | (10) | 110 (68%) | 49 (45%) | 23 (21%) | 14 (13%) |

* TIP related proportion of peritonitis not available in study
