# Supplementary material for: Typhoid intestinal perforation in Francophone Africa, a scoping review
Source: PLOS Glob Public Health. 2024 Mar 29;4(3):e0003056. doi: 10.1371/journal.pgph.0003056 (PMC10980251; doi:10.1371/journal.pgph.0003056)
Supplement: Attachment — Submitted filename: PGPH-D-23-01879_reviewrs comments.docx [file pgph.0003056.s003.docx]

PGPH-D-23-01879

Typhoid Intestinal Perforation in Francophone Africa, a Scoping Review

This is a very relevant and important work with high relevance to public health given that typhoid disease burden continues to remain high in LMICs. Countries like Malawi have set a wonderful example by introducing TCV into their national immunization programme and this valuable work by the authors can serve as a pivotal catalyst for other African countries, especially the Francophone settings, in introducing the vaccine as well. Overall, a well written manuscript. Please find some minor comments to consider.

Abstract

- Lines 35-36: Can this be rephrased for more clarity? – Consider: Currently, data on TIP in Western databases are primarily from countries that speak English, likely due to non-English publication and citation biases from non-English settings.
- Line 36: Removed ‘presumed’
- Lines: 39-10: “..to investigate peritonitis, non-traumatic ileal perforation, and typhoid fever in Francophone African countries” – this is unclear. Aren’t these your MeSH search terms. Please rephrase.
- Line 42: Remove ‘traditional’

Introduction

- Line 66: reads well as ‘surgical condition’
- Lines 86-87: Decision making and public health policy related to? I understand this is related to TCV introduction – please specify.
- It would be good to make a mention of how many countries are Francophone in Africa in the Intro – what % of the population in Africa is Francophone?
- Line 89: Isn’t it ‘incidence’ of TIP?
- Line 90-91: “..for use of TCV in children and its potential to reduce the incidence of this life-threatening surgical condition.”

Materials and methods:

- Line 97: Replace ‘a’ with ‘the’.
- Line 98: Remove ‘broad’
- Line 104: Local health leaders – can you be more specific?

Results:

- Figure 1: The last box showing ‘studies included in review’ = 32. Citation chasing was separate 13 articles – if that was the case it does not sum up to 32. Kindly clarify and make it clear in the flowchart – it is confusing for the reader.
- Supplementary table 1: replace ‘study years’ with ‘study period’; if % for TIP was not available, indicate the same a footnote for the table,
- In Figure 2, it should read as ‘country in search with no relevant data available’? Kindly check and clarify.
- Line 134: Remove ‘old’
- This is perhaps beyond the purview of this scoping review—but were you able to look into if there was documentation of a preceding culture confirmed typhoid in these studies with cases of TIP? I think this is important to be mentioned either in methods or discussion, given that blood culture remains the gold standard for diagnosis and a lot many times false positives are common with other tests used in the Dx of typhoid fever, especially in low-income settings.

Discussion:

- In first para of discussion, would you like to add a sentence on specific typhoid disease burden in this region, as this will add to the case of TIP burden in this region? Also, do these regions have access to TCV within their national immunization programme? These are important to set the background for the burden of TIP and its implications which have been well described in the further paragraphs.
- Line 197: Expand Gavi as it has not been expanded before.
- Line 202: Six countries where?
- Line 231: I just saw that that the blood culture aspect that I mentioned above has been addressed here – great! But would be good if you can say if you looked into this aspect during your review and how many such studies reported the same – perhaps challenging but worth mentioning.
