# Supplementary material for: Typhoid intestinal perforation in Francophone Africa, a scoping review
Source: PLOS Glob Public Health. 2024 Mar 29;4(3):e0003056. doi: 10.1371/journal.pgph.0003056 (PMC10980251; doi:10.1371/journal.pgph.0003056)
Supplement: Attachment — Submitted filename: Francophone TIP-Rebuttal Letter.docx [file pgph.0003056.s004.docx]

26 Dec 2023

Dear PLOS Global Public Health Reviewers,

We would like to express our gratitude for the review of our manuscript, “Typhoid Intestinal Perforation in Francophone Africa, a Scoping Review” (PGPH-D-23-01879). We appreciate the insightful comments that we agree will make our manuscript stronger and clearer. Below are the responses in red to each individual reviewer’s comment.

Reviewer #1: This is a very relevant and important work with high relevance to public health given that typhoid disease burden continues to remain high in LMICs. Countries like Malawi have set a wonderful example by introducing TCV into their national immunization programme and this valuable work by the authors can serve as a pivotal catalyst for other African countries, especially the Francophone settings, in introducing the vaccine as well. Overall, a well written manuscript. Please find some minor comments to consider.”

Thank you for your kind comments regarding our manuscript. As you have identified, we hope the data from this manuscript will expand the introduction of TCVs into the most vulnerable of countries, including many Francophone African countries. We appreciate your comments and have addressed them individually below.

Abstract

- Lines 35-36: Can this be rephrased for more clarity? – Consider: Currently, data on TIP in Western databases are primarily from countries that speak English, likely due to non-English publication and citation biases from non-English settings.
  - This sentence has been reworded to improve clarity.
- Line 36: Removed ‘presumed’
  - The word has been removed.
- Lines: 39-10: “..to investigate peritonitis, non-traumatic ileal perforation, and typhoid fever in Francophone African countries” – this is unclear. Aren’t these your MeSH search terms. Please rephrase.
  - We have added quotations around peritonitis, non-traumatic ileal perforation, and typhoid and clarified that these phrases were the search terms used for the scoping review.
- Line 42: Remove ‘traditional’
  - The word has been removed.

Introduction

- Line 66: reads well as ‘surgical condition’
  - We changed “surgical disease” to “surgical condition” in line 73.
- Lines 86-87: Decision making and public health policy related to? I understand this is related to TCV introduction – please specify.
  - We clarified this by specifically stating that this data will impact decision making and public health policy regarding TCV introduction.
- It would be good to make a mention of how many countries are Francophone in Africa in the Intro – what % of the population in Africa is Francophone?
  - We have included this information in line 87, at the beginning of the fourth paragraph.
- Line 89: Isn’t it ‘incidence’ of TIP?
  - You are correct. We have changed the word prevalence to incidence.
- Line 90-91: “..for use of TCV in children and its potential to reduce the incidence of this life-threatening surgical condition.”
  - We have updated this sentence to reflect this change.

Materials and methods:

- Line 97: Replace ‘a’ with ‘the’.
  - The word was replaced.
- Line 98: Remove ‘broad’
  - This word was removed.
- Line 104: Local health leaders – can you be more specific?
  - Local health leaders was a broad group that included health librarians, medical doctors, along with nursing staff. We felt that it was simpler to reflect all these professions using the broader term of local health leaders.

Results:

- Figure 1: The last box showing ‘studies included in review’ = 32. Citation chasing was separate 13 articles – if that was the case it does not sum up to 32. Kindly clarify and make it clear in the flowchart – it is confusing for the reader.
  - Only 19 articles were reviewed from the traditional databases, resulting in 32 total studies included in the overall review. I have added a line to the final box saying “from traditional databases (n = 19)” to clarify.
- Supplementary table 1: replace ‘study years’ with ‘study period’; if % for TIP was not available, indicate the same a footnote for the table,
  - The table has been updated.
- In Figure 2, it should read as ‘country in search with no relevant data available’? Kindly check and clarify.
  - The figure has been updated.
- Line 134: Remove ‘old’
  - The word has been removed.
- This is perhaps beyond the purview of this scoping review—but were you able to look into if there was documentation of a preceding culture confirmed typhoid in these studies with cases of TIP? I think this is important to be mentioned either in methods or discussion, given that blood culture remains the gold standard for diagnosis and a lot many times false positives are common with other tests used in the Dx of typhoid fever, especially in low-income settings.
  - This is further addressed in the discussion below.

Discussion:

- In first para of discussion, would you like to add a sentence on specific typhoid disease burden in this region, as this will add to the case of TIP burden in this region? Also, do these regions have access to TCV within their national immunization programme? These are important to set the background for the burden of TIP and its implications which have been well described in the further paragraphs.
  - We have not included a statistic regarding typhoid disease burden in this region as we believe current data underestimates the true burden due to a lack of diagnostics in the country. In order to address this, we propose the use of TIP burden as a possibly more accurate surrogate for estimating typhoid burden in paragraph five of the discussion.
  - Regarding access to TCV, this is addressed in the fourth paragraph of the discussion as no Francophone African countries have introduced TCV into their national immunization program.
- Line 197: Expand Gavi as it has not been expanded before.
  - We have added a description of Gavi’s work.
- Line 202: Six countries where?
  - These are the only six countries worldwide that have introduced TCV which has been updated in the discussion.
- Line 231: I just saw that that the blood culture aspect that I mentioned above has been addressed here – great! But would be good if you can say if you looked into this aspect during your review and how many such studies reported the same – perhaps challenging but worth mentioning.
  - We have added that only 3 studies relied on blood culture confirmation.

Reviewer #2: Excellent scoping review on a critical topic of preventable morbidity and mortality: typhoid intestinal perforation (TIP). The authors provide a scoping review of literature with data on TIP in Francophone African countries, and make a specific effort to highlight articles that are not in English. Their results confirm the need for this type of effort -- 22 of 32 manuscripts are in French, and typically left out of the english-speaking academic arena. These data provide meaningful richness to understanding the massive burden and downstream impact of disease - from damage control surgery to ileostomies to social stigma to catastrophic financial expenditure - that is largely preventable with adequate vaccination programs. The authors' discussion is particularly strong, weaves a coherent narrative and serves as potent advocacy for uptake of vaccination programs.

The methods the authors use a well known and accepted set of guidelines for a scoping review. They additionally performed citation chasing, which is important in these types of reviews and contributed/enhanced their yield significantly.

Results: Their figures and tables are clean and clear.

Discussion: As above, excellent contextualization of study findings within the broader African literature on this topic, the disease management challenges in low-resource environments, policy and vaccination landscapes. Limitations are sincere and well presented.

Thank you for the kind comments. We are pleased that we were able to appropriately convey the massive burden typhoid and TIP has upon patients along with the ability to prevent this deadly disease with the use of improved sanitation and available vaccines.

Thank you for the considerable time and effort spent reviewing our manuscript. We believe that the comments from the reviewers have strengthened the manuscript and have now resubmitted it for consideration. Thank you very much and we look forward to hearing your response soon.

Sincerely,

Leah Sukri, MD

Center for Vaccine Development and Global Health

University of Maryland School of Medicine

685 W. Baltimore Street

Baltimore, MD 21201

[Leah.sukri@som.umaryland.edu](mailto:Leah.sukri@som.umaryland.edu)
